# Study on the cellular internalization mechanisms and *in vivo* anti-bone metastasis prostate cancer efficiency of the peptide T7-modified polypeptide nanoparticles

**DOI:** 10.1080/10717544.2019.1709923

**Published:** 2020-01-08

**Authors:** Yongwei Gu, Xinmei Chen, Haiyan Zhang, Heyi Wang, Hang Chen, Sifan Huang, Youfa Xu, Yuansheng Zhang, Xin Wu, Jianming Chen

**Affiliations:** a Department of Pharmacy, Fujian University of Traditional Chinese Medicine, Fuzhou, China;; b School of Pharmacy, Second Military Medical University, Shanghai, China;; c Department of Pharmacy, Inner Mongolia Medical University, Huhhot, China;; d Shanghai Wei Er Biopharmaceutical Technology Co., Ltd, Shanghai, China

**Keywords:** Gene therapy, bone-metastases prostate cancer, cellular internalization mechanisms, anti-tumor effect, administration safety

## Abstract

Bone-metastasis prostate cancer (BMPCa)-targeting gene therapy is gaining increasing concern in recent years. The peptide T7-modified polypeptide nanoparticles for delivery DNA (CRD-PEG-T7/pPMEPA1) was prepared as our previous study. However, the feasibility of CRD-PEG-T7/pPMEPA1 for BMPCa treatment, the mechanisms underlying cellular uptake, anti-BMPCa effect, and administration safety requires further research. LNCaP cells treated with endocytosis inhibitors and excessive T7 under different culture condition were carried out to investigate the mechanisms of cellular uptake of the CRD-PEG-T7-pPMEPA1. A transwell assay was applied to evaluate the cell migration ability. Besides, the tumor volume and survival rates of the PCa xenograft mice model were recorded to estimate the anti-tumor effect. In addition, the weight profiles of the PCa tumor-bearing mice, the blood chemistry, and the HE analysis of visceral organs and tumor was conducted to investigate the administration safety of CRD-PEG-T7/pPMEPA1. The results showed that PCa cellular uptake was decreased after treating with excessive free T7, endocytosis inhibitors and lower incubation temperature. Besides, CRD-PEG-T7/pPMEPA1 could inhibit the LNCaP cells chemotaxis and tumor growth. In addition, the survival duration of the PCa tumor-bearing mice treating with CRD-PEG-T7/pPMEPA1 was significantly prolonged with any systemic toxicity or damage to the organs. In conclusion, this research proposes a promising stratagem for treatment BMPCa by providing the biocompatible and effective carrier for delivery DNA therapeutic agents.

## 1. Introduction

Prostate cancer (PCa) is the second death-leading malignancy in American men (Siegel et al., 2019). And about 90% of patients with PCa develop BMPCa, bringing intolerable complications and uprising mortality (Bubendorf et al., 2000; Halabi et al., 2016; Nakazawa et al., 2017; Nyquist & Nelson, 2017). There are some side effects on conventional aggressive surgery and chemotherapy. Especially, bone-targeting agents including docetaxel ± dadatinib, atrasentan were failed in preclinical and phase II trial (Miyahira & Soule, 2019). Recently, gene therapy for metastatic prostate cancer gain desired development (Gu et al., 2014; Wu et al., 2014).

For prostate cancer, TGF-β signaling pathway is closely related to cell proliferation, which inhibits tumor cells growth in early-stage while promotes bone metastasis in later stages (Donkor et al., 2011). Studies have demonstrated that TGF-β could upregulate the expression of the prostate transmembrane protein androgen induced-1 (PMEPA1) which was encoded by the androgen-regulated gene expressed in PCa (Singha et al., 2010; Watanabe et al., 2010; Amalia et al., 2019). Furthermore, clinical statistics revealed that low expression of PMEPA1 led to bone metastases and dropped survival. In BMPCa patients, PMEPA1 was decreased that regulates the tumor cells invasion, proliferation, and metastasis (Fournier et al., 2015; Xu et al., 2019). 20–25% of metastatic PCa harbor defects in DNA repair genes (Athie et al., 2019). And gene therapy for PCa is gaining great achievements through direct delivering DNA or RNA into cancer cells.

We previously reported an effective BMPCa cells-targeting cationic polypeptide gene carrier modified with peptide (T7, amino acid sequence: HAIYPRH) that could directly transfer plasmid DNA (pDNA, pPMEPA1) into PCa cells, actively targeting to over expressing transferrin receptors (TfR) in PCa cells (Lu et al., 2018). In addition, the polypeptide, (arginine (R)-aspartic (D) acid peptide (RRRRRRRCDDDDDD)) known as R7D6 synthesized by F-mocsolid-phase synthesis method could target to bone guiding with the aspartic acid short peptide sequence (de Kroon et al., 2017). Then, under oxidizing conditions, R7D6 and L-Cys·HCL react to form CRD (C: L-Cys, R: arginine; D: aspartic acid) that possessed disulfide bonds. The polymers with disulfide bonds have the properties of biodegradation, high stability, low biotoxicity, high transfection efficiency, and rapid intracellular drug release (Lin et al., 2006; Kim et al., 2010). Then the CRD was modified with peptide T7 to construct the final tumor-targeting gene delivery vector CRD-PEG-T7 (Lu et al., 2018). Interestingly, this vector could form nanoparticles with pPMEPA1 at a certain N/P ratio. Therefore, we prepared CRD-PEG-T7 and pPMEPA1 to form the CRD-PEG-T7/pPMEPA1 complexes. The cunning characteristics were as follows: (1) the cationic polymer material could highly encapsulate gene; (2) the polypeptide and the nanoparticles modified with peptide T7 could actively target to bone and PCa cells; and (3) pPMEPA1 delivered by CRD-PEG-T7 could enhance anticancer effects precisely.

Though the dual targetting vector of CRD-PEG-T7/pPMEPA1 was prepared in our previous study. However, there remain elusion in terms of endocytosis mechanisms, anti-tumor effect and safety evaluation. In this study, the endocytosis inhibitors were used to illustrate the transmembrane mechanisms of the complexes. And, a transwell assay was applied to study the inhibition effect of the modified nanoparticles in tumor cell migration. Besides, pharmacodynamics was carried out to investigate the *in vivo* anti-tumor effect. Finally, the toxicity of systemic and organs was tested to evaluate the administration safety of the CRD-PEG-T7/pPMEPA1.

## 2. Material and methods

### 2.1. Materials

The materials used in this study were as follows: Arginine-aspartic acid peptide monomer (sequence: RRRRRRRCDDDDDD, R7D6) and peptide T7 (sequence: HAIYPRH) (Ontores Biotechnologies, Zhejiang, People’s Republic of China); NHS-PEG-MAL (α-maleimide-ω-N-hydroxysuccinimidyl polyethyleneglycol, MW 3500, Nektar Therapeutics, Huntsville, AL, USA); pPMEPA1, YOYO1-pPMEPA1 (General Biosystems, Anhui, People’s Republic of China); Fetal bovine serum (FBS), RPMI medium 1640 basic, Trypsin and 1% Pen Strep (Thermo Fisher Scientific, Waltham, MA). The other chemicals and reagents were of analytical grade.

### 2.2. Cells and cell culture

Prostate carcinoma cells (LNCaP, American Type Culture Collection, Manassas, VA, USA) were cultured in RPMI medium 1640 basic containing 10% FBS and 1% Pen Strep under 5% CO_2_ atmosphere at 37 °C. When reaching 80–90% confluence, the cells were trypsinized and resuspended for further use.

### 2.3. Animals

Four-week-old male BALB/c nude mice (18–22 g) purchased from Shanghai SLAC Laboratory Animal Co., Ltd., (Shanghai China) were housed under standard laboratory conditions. All animal protocols complied with the International Ethical Guideline and National Institutes of Health Guidelines on the Care and Use of Laboratory Animals, and with the approval of the Institutional Animal Care and Use Committee of Fujian University of Traditional Chinese Medicine.

### 2.4. Synthesis of polypeptide gene carrier

Polypeptide gene carrier was successfully synthesized by the F-mocsolid-phase synthesis method described as our earlier study (Lu et al., 2018). Briefly, R7D6 monomers (arginine–aspartic acid peptide, sequence CRRRRRRRCDDDDDD) dissolved in 10 mL distilled water, the l-cysteine hydrochloride monohydrates (Cys) were added in the mixture at the molar ratios of 5:1. Followed by, the system was added with 1% H_2_O_2_ of 0.5 mL dropwise. After 12 h, the acid peptide linked with disulfide bonds known as CRD was extracted and purified. Then, CRD were reacted with NHS-PEG-MAL (MW: 3400) at the molar ratio of 1:10 in distilled water for 6 h to produce CRD-PEG-MAL. Finally, the conjugate was reacted with Cys-T7 at a molar ratio of 1:5 in distilled water for 6 h to form the final product CRD-PEG-T7. All the reactions were conducted under room temperature.

### 2.5. Preparation of the peptide T7-modified polypeptide nanoparticles

The CRD-PEG-T7 solution and pPMEPA1 (2 μg) with N/P ratio of 15 was vortexed for 30 s. The samples were then incubated for 30 min at room temperature to obtain CRD-PEG-T7/pPMEPA1. In addition, R7D6/pPMEPA1, CRD-PEG-T7/YOYO1-pPMEPA1, and R7D6/YOYO1-pPMEPA1 were prepared with the same method. The particle size and zeta potential of CRD-PEG-T7/pPMEPA1 was measured using a Zeta-sizer Nano ZS90 (Malvern, USA). The morphology was visualized by transmission electron microscopy (TEM, 100CXII, Japan).

### 2.6. Internalization mechanisms

Cells cultured with endocytic inhibitors or excessive T7 at different temperature were applied to investigate the cellular uptake mechanisms of CRD-PEG-T7/pPMEPA1 (Wu et al., 2014). LNCaP cells suspensions were incubated into a 24-well plate at a density of 2 × 10^5^ cells per well for 24 h. Then the cell culture medium was replaced with CRD-PEG-T7/YOYO1-pPMEPA1 at 4 °C, CRD-PEG-T7/YOYO1-pPMEPA1 at 37 °C, or CRD-PEG-T7/YOYO1-pPMEPA1 with excessive free T7 (100 mM) at 37 °C. After incubation for 1 h, LNCaP cells were exposed to a fluorescent microscope (Leica Microsystems, Wetzlar, Germany) to monitor the cellular uptake. In addition, the cells uptake rate was detected by flow cytometry (NIKON, Japan).

Besides, LNCaP cells suspensions were incubated into a 24-well plate at a density of 2 × 10^5^ cells per well for 24 h. Then the cell culture medium was replaced with the fresh serum-free RPMI 1640 containing colchicine (10 nM), filipin (5 mg/mL), PhAsO (1 mM), polylysine (5 mg/mL) and excessive T7 (100 mM) for 10 min at 37 °C, respectively. Subsequently, the cells were washed with PBS and treated with 100 nM CRD-PEG-T7/YOYO1-pPMEPA1 complexes or R7D6-YOYO1-pPMEPA1 for another 1 h at 37 °C. The cellular uptake was then imaged by fluorescent microscope (Leica Microsystems, Wetzlar, Germany). The cells cultured only with CRD-PEG-T7/YOYO1-pPMEPA1 or R7D6-YOYO1-pPMEPA1 as control. For quantitative analysis, the cells were treated with 1% Triton X-100 and centrifuged at 3000 rpm for 15 min. The fluorescence intensity of the YOYO1 in the supernatant was determined by a microplate fluorometer (LS 55, PerkinElmer Inc, Waltham, MA, USA). Besides, the protein concentration in cell lysis fluid was quantified using Bradford assay (Beyotime) with bovine serum albumin as a protein standard. The relative uptake efficiency (RUE) was calculated as the following formula (Huang et al., 2009).

RUE = (experimental fluorescent intensity/mg protein)/(control fluorescent intensity/mg protein)×100%

### 2.7. Cells chemotaxis

Transwell assay was applied to evaluate the LNCaP migration after the cells treated with CRD-PEG-T7/pPMEPA1, CRD-PEG-T7 and R7D6/pPMEPA1 (Zhang et al., 2016). Briefly, LNCaP cells cultured with FBS-free RPMI medium 1640 basic for 24 h and then treated with CRD-PEG-T7/pPMEPA1, R7D6/pPMEPA1 and CRD-PEG-T7 for another 4 h. Then the cells were cultured with FBS-free medium for another 24 h and digested. Followed by, the cells were added into the insert of Transwell (Matrigelfree polycarbonate membranes, 24-well plant, pore size 8 µm), while the bottom champers with RPMI medium 1640 basis containing 10% FBS was used as cell chemotaxis. After 48 h incubation, the cells in the top-side were removed and the cells migrated to the bottom side of the insert were fixed with 4% paraformaldehyde and stained with violet crystal. Subsequently, the transwell insert member was visualized by fluorescence microscopy and the migrated cells were counted by software Image J.

### 2.8. *In vivo* anti-tumor effect

A xenograft tumor model was established by injecting 2 × 10^6^ LNCaP cells into the tibia of 4-week-old male BALB/c nude mice. When the tumor volume reached about 100 mm^3^, the mice were randomized into 4 groups and respectively injected with 0.2 mL normal saline (control), CRD-PEG-T7, R7D6/pPMEPA1, and CRD-PEG-T7/pPMEPA1 through tail vein at days 1, 3, 5, 7, and 11 (Fisher et al., 2002). The first injection day was set as day 1. The dose was equivalent to 50 μg/kg of pPMEPA1. Tumor volume ($v=\frac{ab^{2}}{2},$ v: the tumor volume, *a*: the longest diameter of tumor, *b*: the shortest diameter of tumor) was measured every other day. The survival rates were recorded after administration and the tumors were surgically excised and weighted when the mice died.

### 2.9. Systemic toxicity

The systemic toxicity of CRD-PEG-T7/pPMEPA1 was evaluated by the tumor-bearing mice weight changes and routine blood examination. The xenograft tumor model of BALB/c nude mice was randomized into 4 groups: normal saline (control), CRD-PEG-T7, R7D6/pPMEPA1, and CRD-PEG-T7/pPMEPA1. Then the mice were respectively injected with 0.2 mL of the different samples through tail vein at days 1, 3, 5, 7, and 11. Body weight was measured every other day after administration. After the last day of administration, orbital blood was sampled and subjected to a routine blood test. The neutrophile (Gran), red blood cell (RBC), Hemoglobin blood (HGB), platelet (PLT), and white blood cell (WBC) count were measured through a blood cell analyzer (ADVIA2120, Siemens, Germany).

### 2.10. Histological (HE) analysis

HE analysis was carried out to study the effect of CRD-PEG-T7/pPMEPA1 on organs of the heart, liver, spleen, lung, kidneys, and tumor. Briefly, xenograft tumor model of BALB/c nude mice was randomized into 4 groups (*n* = 6) and respectively injected with 0.2 mL normal saline (control), CRD-PEG-T7, R7D6/pPMEPA1, and CRD-PEG-T7/pPMEPA1 through tail vein at days 1, 3, 5, 7, and 11. After the last day of administration, the mice were euthanized and the organs of the heart, liver, spleen, lung, kidneys, and tumor were harvested. Then the samples were stained with hematoxylin and eosin (H & E) under the guidance of manufacturers’ standard protocol and imaged using a light microscope (Leica, Germany).

### 2.11. Statistical analysis

The results were represented as a mean of at least three experiments with the corresponding standard deviation (SD). Statistical data were analyzed using SPSS software version 18.0 and a statistically significant difference was denoted by the difference probability level (*p* < .05). *t*-Test and least-significant different (LSD) were used to analyze the statistical data.

## 3. Results

### 3.1. Characterization of CRD-PEG-T7/pPMEPA1

**Figure 1.**
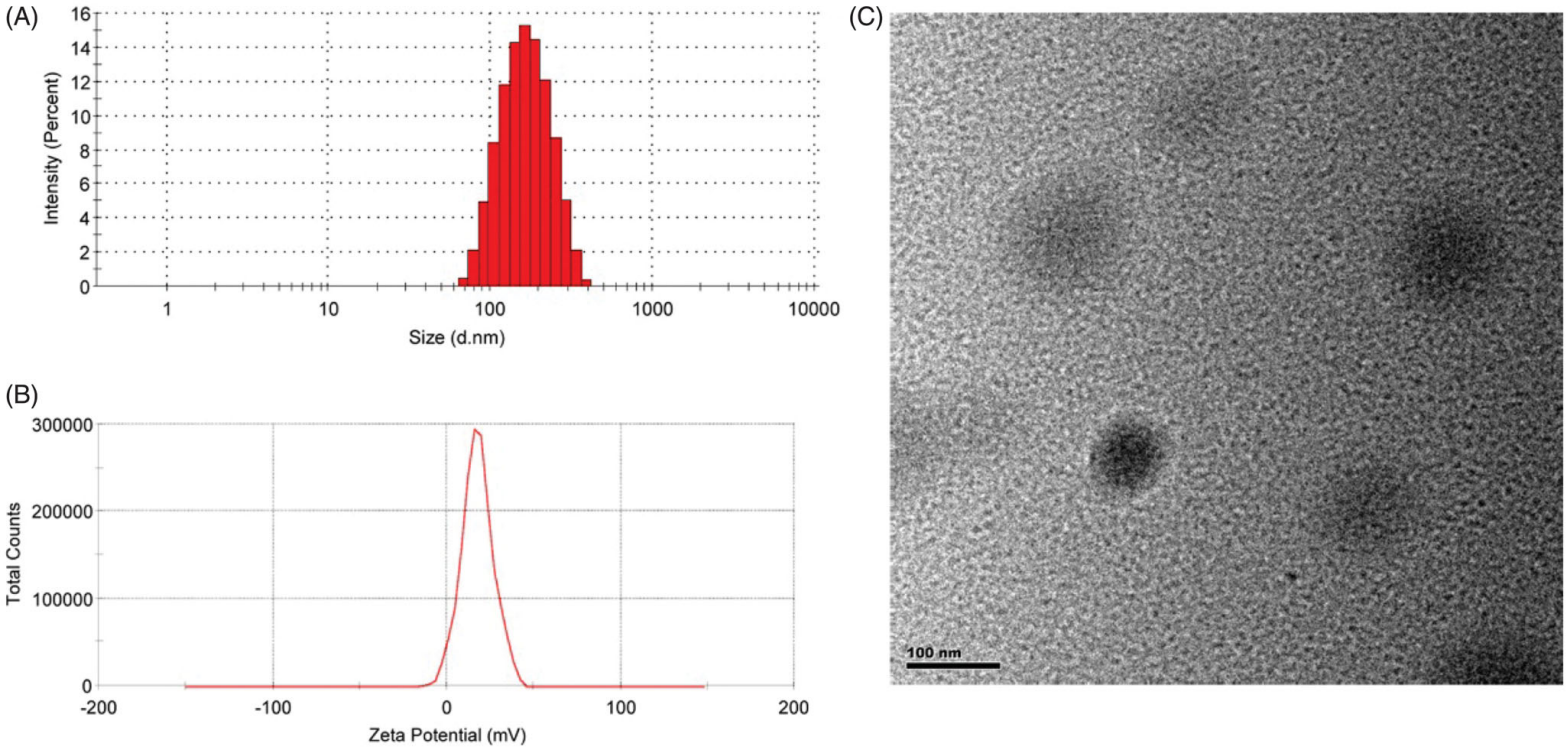
The size distribution (A); zeta potential (B); and morphology (C) of the CRD-PEG-T7/pPMEPA1.

The particle size, zeta potential, and morphology of the prepared CRD-PEG-T7/pPMEPA1 are shown in Figure 1. The size and polydispersity index (PDI) were 152.9 ± 4.30 and 0.126 ± 0.006, respectively (Figure 1(A)). And the zeta potential was 17.6 ± 0.62 (Figure 1(B)). As shown in Figure 1(C), the morphology of CRD-PEG-T7-pDNA was mostly spherical and the size distribution was in accordance with the results of laser scattering technique.

### 3.2. Internalization mechanisms

**Figure 2.**
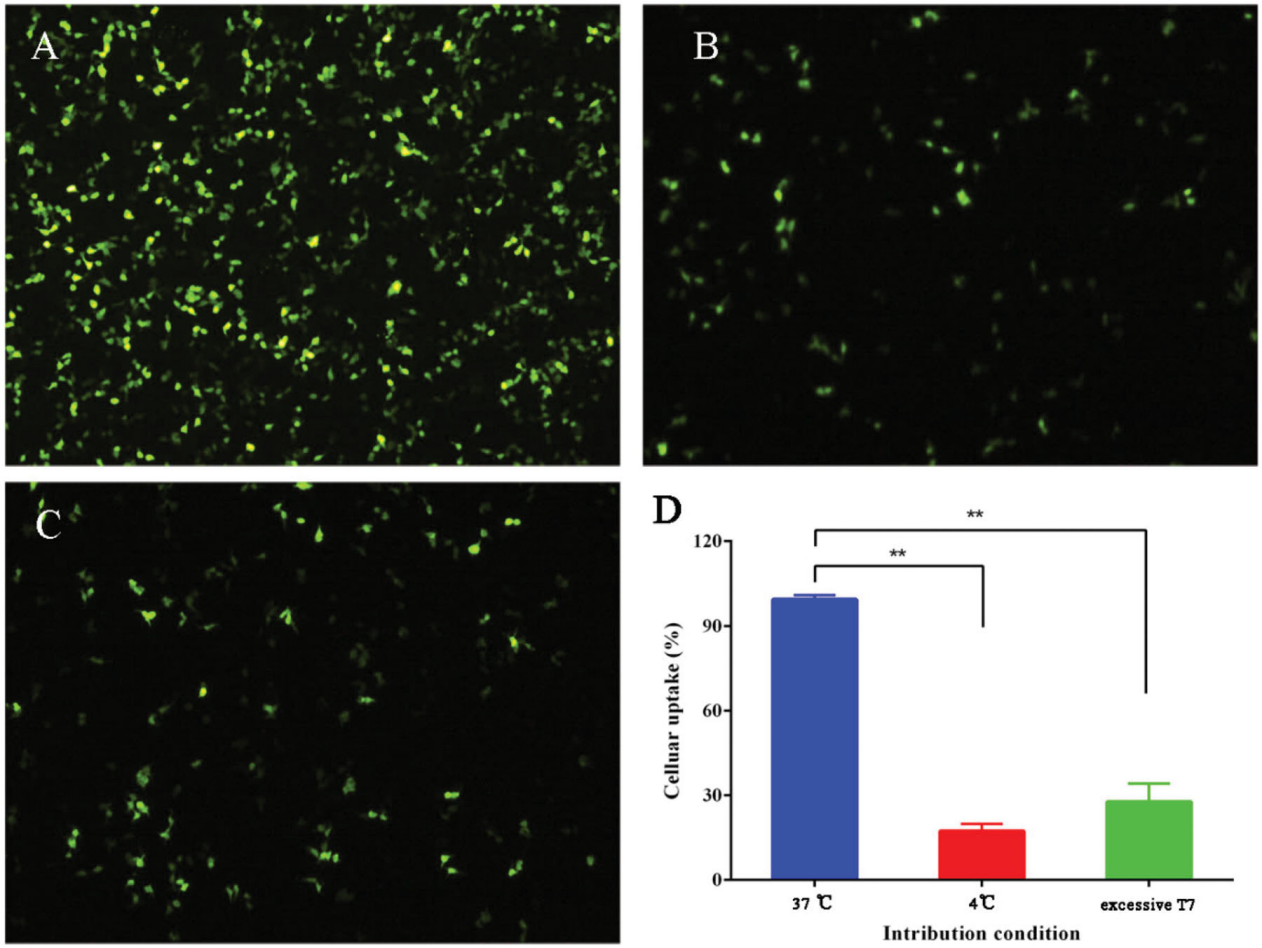
The cellular uptake of CRD-PEG-T7/YOYO1-pPMEPA1 at 37 °C (A), 4 °C (B), with excessive free T7 at 37 °C (C), and the quantitative evaluation of the cellular uptake in the different intribution condition (D).

Cell uptake at different incubation conditions is shown in Figure 2. The cellular uptake of CRD-PEG-T7/YOYO1-pPMEPA1 at 37 °C was significantly high than that at 4 °C (Figure 2(A,B)). In addition, cellular uptake in the group with excessive free T7 was lower compared with that in the CRD-PEG-T7/YOYO1-pPMEPA1 group at 37 °C (Figure 2(B,C)). The results of cellular uptake were consistent with the fluorescent images.

**Figure 3.**
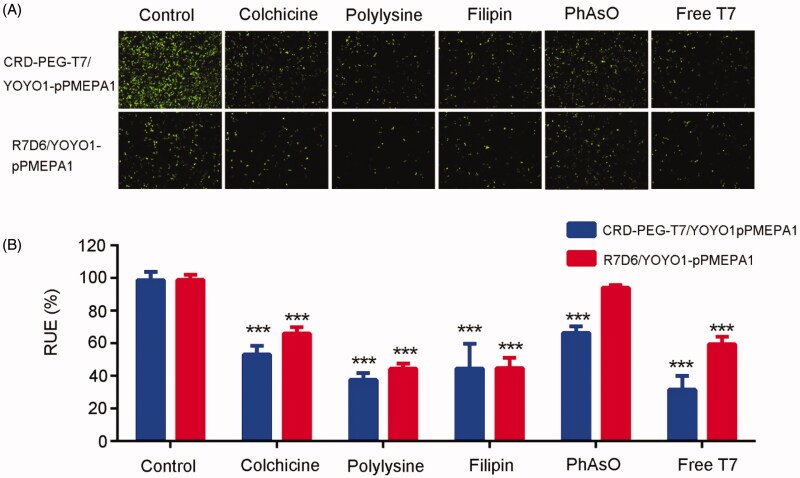
Qualitative and quantitative evaluation of cellular uptake of CRD-PEG-T7/YOYO1- pPMEPA1 and R7D6/YOYO1-pPMEPA1: The fluorescence intensity (A) and RUE (B) of the different groups incubated with various cell uptake inhibitors. (*** means *p* < .001).

Figure 3 was the results of cell uptake effect of the LNCaP cells pretreated with different inhibitors. The fluorescence intensity in cells treated with inhibitors was lower than that in the control group (CRD-PEG-T7/YOYO1-pPMEPA1) in different degrees as shown in Figure 3(A). Similarly, the cellular uptake of R7D6/YOYO1-pPMEPA1 was inhibited by the most inhibitors except for the PhAsO. As shown in Figure 3(B), the quantitative analysis of RUE consistent with the results of the fluorescence microscope. The RUE of CRD-PEG-T7/YOYO1-pPMEPA1 group treated with colchicine, cationic polylysine, filipin, PhAsO, and excessive T7 was 53.17 ± 5.30%, 37.66 ± 4.17%, 44.56 ± 15.30%, 66.36 ± 4.05%, 31.60 ± 8.41%, while that of R7D6/YOYO1-pPMEPA1 group were 66.07 ± 3.73%, 44.42 ± 3.23%, 44.82 ± 6.23%, 91.37 ± 5.11%, 59.61 ± 4.38%, respectively (the RUE value of CRD-PEG-T7/YOYO1-pPMEPA1 and R7D6/YOYO1-pPMEPA1 was both 100%).

### 3.3. Cells chemotaxis

**Figure 4.**
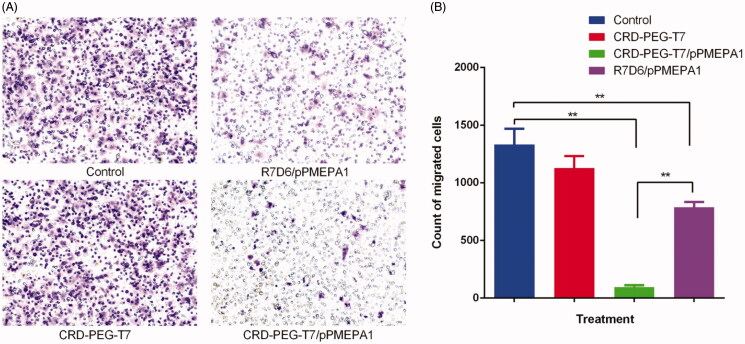
LNCaP cells migration assay using transwell assay. (A) the microphotograph of LNCaP cells attached to the transwell assay membrane; (B) the migrated cells count in groups of control, CRD-PEG-T7, CRD-PEG-T7/pPMEPA1, and R7D6/pPMEPA1 (** means *p* < .01).

The migration LNCaP cells stained by crystal violet and the migration cell counts were shown in Figure 4. The results of microscopic photographs and migration cells counts indicated that the effect of migration of LNCaP cells treated with CRD-PEG-T7/pPMEPA1 was significantly inhibited compared with the control group (*p* < .01). Besides, it was significant different in the inhibition effect between CRD-PEG-T7/pPMEPA1 and R7D6/pPMEPA1 (*p* < .01). In addition, the counts of migrated cells of the control, CRD-PEG-T7, R7D6/pPMEPA1, and CRD-PEG-T7/pPMEPA1 were 1333 ± 135, 1128 ± 103, 788 ± 46, and 94 ± 18, respectively.

### 3.4. *In vivo* anti-tumor effect

**Figure 5.**
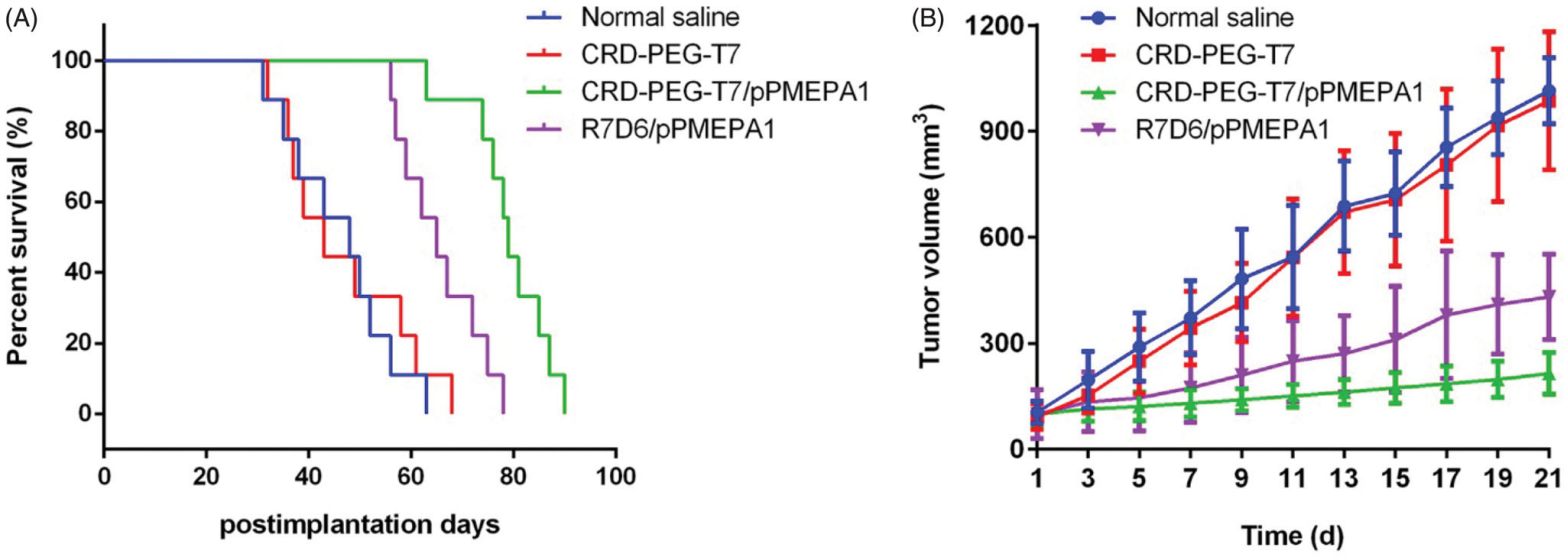
The *in vivo* anti-tumor effect of CRD-PEG-T7/pPMEPA1, R7D6/pPMEPA1, and CRD-PEG-T7 in LNCaP cell-derived tumor-bearing mice model. (A) The Kaplan–Meier survival curve; (B) The tumor volumes.

Nude mice bearing human transplanted prostate cancer were randomly divided into four groups: CRD-PEG-T7, CRD-PEG-T7/pPMEPA1, R7D6/pPMEPA1, and normal saline (as control). The survival duration profiles reflecting the anticancer effect are presented in Figure 5(A) and Table 1. The results indicated that CRD-PEG-T7/pPMEPA1 and R7D6/pPMEPA1 could effectively prolong the mice lifetime and that the antitumor effect of CRD-PEG-T7/pPMEPA1 was superior to that of the unmodified polypeptide nanoparticles (*p* < .5). The mean survival duration of the control, CRD-PEG-T7, CRD-PEG-T7/pPMEPA1, and R7D6/pPMEPA1 were 46.2 days, 47.0 days, 79.2 days, and 60.1 days, respectively. The log-rank analysis showed that the survival duration in the CRD-PEG-T7/pPMEPA1 group was significantly longer compared with the others groups (*p* < .01). As shown in Figure 5(B), the significant suppression in tumor growth was also observed in the group of CRD-PEG-T7/pPMEPA1. Besides, the results of the mean tumor weight were displayed in Table 1 showed that the CRD-PEG-T7/pPMEPA1 performed sustainable suppressive effect on tumor growth.

**Table 1.** The *in vivo* effect of CRD-PEG-T7/pPMEPA1 on tumor-bearing mouse model.

Groups	MST (days)	Median (days)	The average weight of tumors	Compared with normal saline	Compared with CRD-PEG-T7	Compared with R7D6/pPMEPA1
Normal Saline	46.2	48	1.36 ± 0.15	–	–	–
CRD-PEG-T7	47.0	43	1.35 ± 0.26	*p* > .05	–	–
CRD-PEG-T7/pPMEPA1	79.2	79	0.14 ± 0.01	**	**	**
R7D6/ pPMEPA1	60.1	65	0.65 ± 0.19	**	**	–

Note: **p < .01 of the log-rank analysis.

Abbreviation: MST, median survival time.

### 3.5. Systemic toxicity

**Figure 6.**
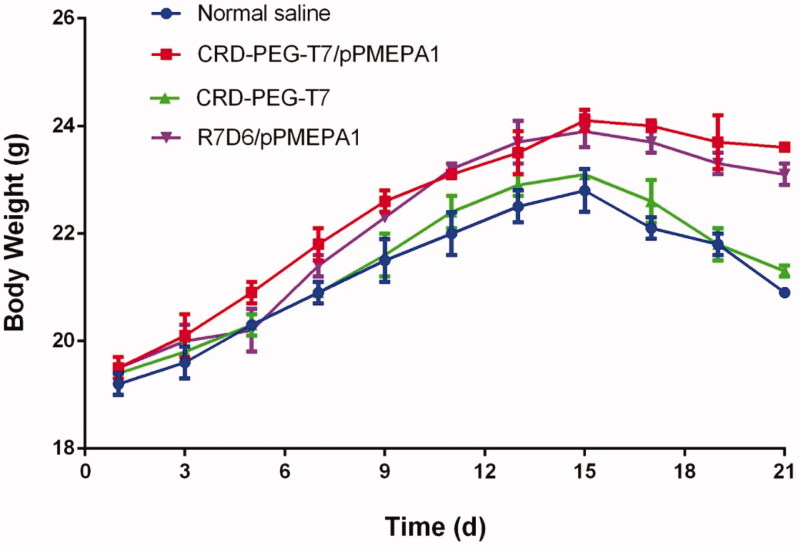
The changes in the bodyweight of PCa cancer tumor-bearing mice over time (*n* = 10).

As shown in Figure 6, the mice weight of the CRD-PEG-T7, CRD-PEG-T7/pPMEPA1, R7D6/pPMEPA1, and control groups was monitored for 21 d. There are no significant differences in the groups of control and CRD-PEG-T7, implying that CRD-PEG-T7 was a safe carrier for gene therapy. In addition, the weight of tumor-bearing mice treated with CRD-PEG-T7/pPMEPA1 and R7D6/pPMEPA1 was heavier than that treated with normal saline and blank carriers. What’s more, the hematological toxicity of tumor-bearing mice treated with CRD-PEG-T7/pPMEPA1 was studied by routine blood examination. As shown in Table 2, the parameters of Gran, RBC, PLT, and WBC were significantly decreased, while the HGB index was up-regulated in the groups of polypeptide nanoparticle loading gene.

**Table 2.** The hematological parameters of the tumor-bearing mice with different treating (*n* = 10).

Groups	Gran (×10^9^/L)	RBC (×10^12^/L)	HGB (g/L)	PLT (×10^9^/L)	WBC (×10^9^/L)
Normal saline	9.69 ± 1.08	7.41 ± 0.25	132.59 ± 7.49	1593.04 ± 352.71	20.72 ± 2.27
CRD-PEG-T7	9.71 ± 0.19	7.44 ± 0.11	135.16 ± 6.92	1590.14 ± 141.79	21.23 ± 2.25
R7D6/pPMEPA1	9.42 ± 1.97**	7.24 ± 1.08**	149.75 ± 9.98**	1264.62 ± 268.32**	18.81 ± 3.20**
CRD-PEG-T7/ pPMEPA1	9.47 ± 2.18**	7.19 ± 0.97**	143.81 ± 10.07**	1413.53 ± 289.05**	19.31 ± 4.21**

Note: **p < .01 of the variance analysis compared with the group of normal saline.

Abbreviations: Gran: neutrophile granulocyte; RBC: red blood cell; HGB: Hemoglobin blood; PLT: platelet; WBC: white blood cell.

### 3.6. Histological (HE) analysis

**Figure 7.**
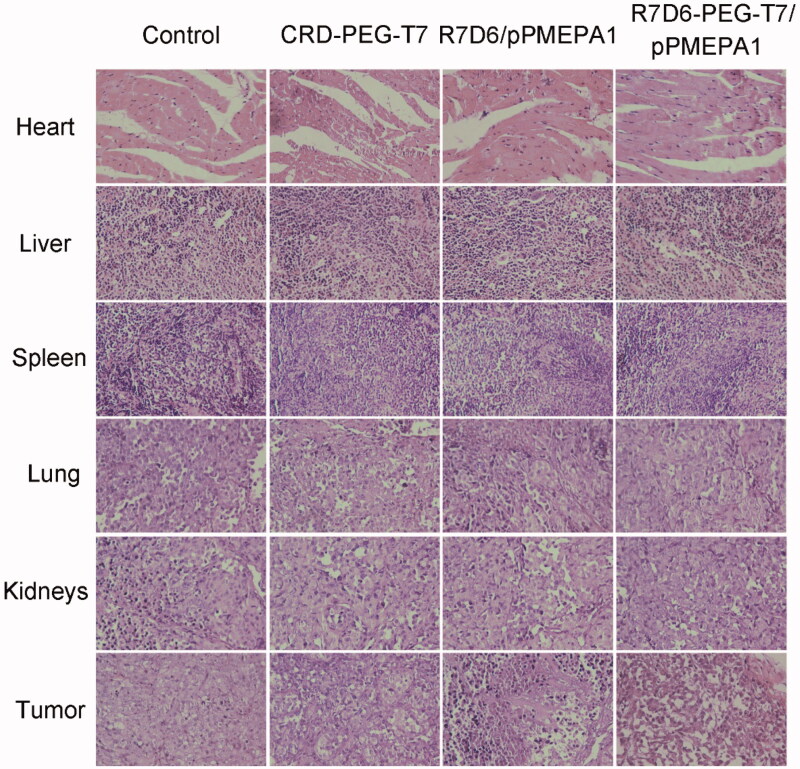
Histopathological photomicrographs of tumor-bearing mice heart, liver spleen, lung, kidneys, and tumor. (×200).

The HE microscopic pictures of the heart, liver, spleen, lung, kidneys, and tumor of mice treated with normal saline, CRD-PEG-T7, R7D6/pPMEPA1, and CRD-PEG-T7/pPMEPA1 are shown in Figure 7. It was obvious that the cells were necrosis, fibrosis with hemorrhage in the tumor tissue treated with CRD-PRE-T7/pPMEPA1 compared with normal saline. Besides, in the four groups, there were no obvious abnormalities, lesions or degenerations in visceral organs, indicating the CRD-PRE-T7/pPMEPA1 did not induce any organotoxicity.

## 4. Discussion

Peptide T7-modified vectors (CRD-PEG-T7) prepared using the F-mocsolid-phase synthesis method was evaluated as gene delivery (CRD-PEG-T7/pPMEPA1) for BMPCa treatment. The morphology of the nanoparticles was uniform with small particle size distribution and positive charge. Our previous reported that the developed CRD-PEG-T7/pPMEPA1 was characteristic of admirable gene transfection and targeting efficacy with excellent biocompatibility (Lu et al., 2018).

LNCaP cells cultured with different incubation condition, excessive T7, and endocytic inhibitors were applied to investigate the cellular uptake mechanisms of CRD-PEG-T7/pPMEPA1. The results of cellular uptake at different incubation temperature indicated that the cellular uptake of CRD-PRG-T7/pPMEPA1 was characterized by energy dependence. Besides, early-stage, excessive T7 could inhibit the cellular uptake of CRD-PEG-T7 competitively. In addition, colchicine inhibits the macropinocytosis and cationic polylysine is an inhibitor to uptake the cationic NPs (Wu et al., 2014; Voltan et al., 2017). The caveolae-mediated process and clathrin-dependent endocytosis was blocked by flipin and PhAsO, respectively (Kim et al., 2007; Liu et al., 2018b). The results showed that the cell uptake of CRD-PEG-T7/YOYO1-pPMEPA1 was proportional to energy and inversely to the presence of T7, which reflected that T7 competitively uptake with CRD-PEG-T7/pPMEPA1. In addition, LNCaP uptake of CRD-PEG-T7/pPMEPA1 was depending on macropinocytosis, caveolae-mediated endocytosis, and clathrin-dependent endocytosis (Figure 3). These results indicated that the mechanisms of receptor and adsorptive-mediated might contribute to the cellular uptake of CRD-PEG-T7/pPMEPA1 (Huang et al., 2009; Wu et al., 2014).

Cell migration assay plays a central role in the evaluation of cancer migration. And the cell culture medium containing FBS often was used as chemokines (Qiang et al., 2019). The results of transwell assay indicated that the CRD-PEG-T7/pPMEPA1 could significantly inhibit the LNCaP migration compared with the unmodified polypeptide nanoparticles R7D6/pPMEPA1, while the blank carrier CRD-PEG-T7 same as control group has no inhibition of migration. The results were consistent with the previous report that the pPMEPA1 could restrain the DU145 cells migration and the reduce gene expression followed by lower tumor progression (Feng et al., 2016; Lu et al., 2018). Besides, the difference between the groups of CRD-PEG-T7/pPMEPA1 and R7D6/pPMEPA1 might be due to the high cell uptake of the nanoparticles modified by the peptide T7 showed in Figure 4.

The results of *in vivo* anti-tumor indicated that CRD-PEG-T7/pPMEPA1 could prolong the median survival time and inhibit the tumor growth with the smallest tumor volume in the human PCa mouse model (Figure 5 and Table 1). The polypeptide nanoparticle modified by the peptide T7 loading pPMEPA1 genes was successfully designed to target tumor and blocked the pathways involved in PCa development, potentially promoting the anti-tumor effect (Fournier et al., 2015; Sharad et al., 2014; Lu et al., 2018).

The results of systemic toxicity showed that CRD-PEG-T7/pPMEPA1 were well biocompatibility. There was no significant fluctuation in body weight among the four groups (Figure 6). However, the humor-bearing mice treated with normal saline and CRD-PEG-T7 were skinny though the weight was similar to the other mice owing to the growing tumor volume (Liu et al., 2018a). Besides, the blood parameters of Gran, RBC, PLT, WBC were decreased compared with the control group (Table 2), indicating that the inflammation and tissue impairment was improved (El-Hag & Clark, 1987; Gay & Felding-Habermann, 2011; Moreel et al., 2014; Paramanathan et al., 2014; Arthur et al., 2018). What’s more, the up-regulated HGB demonstrated that the anemia symptoms in tumor-bearing mice were effectively improved after treating with CRD-PEG-T7/pPMEPA1 (Beer et al., 2006). Furthermore, the pathological HE analysis of tumor indicated (Figure 6) that CRD-PEG-T7/pPMEPA1 could enhance cell necrosis, fibrosis with hemorrhage (Li et al., 2018; Yang et al., 2018). The HE photographs of heart, liver, spleen, lung and kidney indicated that CRD-PEG-T7/pPMEPA1 did not induced any physiological function impact compared with the control group (Lu et al., 2018). The results implied that the DNA delivery vectors of CRD-PEG-T7 are a safe and effective treatment agent for BMCPa.

## 5. Conclusion

To the best of our knowledge, this is the first study on the PCa cells uptake mechanisms, anticancer effect *in vivo* and safety evaluation of the peptide T7 modified-polypeptide nanoparticles for delivery gene (CRD-PEG-T7/pPMEPA1). The LNCaP cells uptake of T7 modified NPs by endocytic processes, depending on macropinocytosis, caveolae, clathrin, the cationic vectors nanoparticles. Besides, CRD-PEG-T7/pPMEPA1 could significantly inhibit LNCaP cells migration in vitro. In addition, the prepared gene complex performed significant anti-tumor effect *in vivo* without inducing impairment to normal tissues. In conclusion, this research proposes a prototype of the safe and effective carrier for delivery DNA to PCa cells, which could be a promising stratagem for treating BMPCa.
